# Diagnostic significance of salivary and glandular siglec-5 in Sjögren disease and non-Sjögren *sicca*

**DOI:** 10.1007/s00011-026-02188-8

**Published:** 2026-02-26

**Authors:** Fernanda Luiza Araújo de Lima Castro, Laiz Fernandes Mendes Nunes, Fernanda Aragão Felix, Sicília Rezende Oliveira, José Alcides Almeida de Arruda, Victor Zanetti Drumond, Lucas Guimarães Abreu, Anna Christina Higino Rocha, Camila Munayer Lara, Harim Tavares Dos Santos, Maurício Augusto Aquino de Castro, Gilda Aparecida Ferreira, Leandro Augusto Tanure, Débora Cerqueira Calderaro, Tarcília Aparecida Silva, Sílvia Ferreira de Sousa

**Affiliations:** 1 Department of Oral Surgery, Pathology and Clinical Dentistry, School of Dentistry, Universidade Federal de Minas Gerais, Av. Pres. Antônio Carlos, 6627, Sala 3204,, Belo Horizonte, Minas Gerais CEP: 31.270-910 Brazil; 2 Department of Oral Diagnosis and Pathology, School of Dentistry, Universidade Federal do Rio de Janeiro, Rio de Janeiro, Brazil; 3 Department of Child and Adolescent Oral Health, School of Dentistry, Universidade Federal de Minas Gerais, Belo Horizonte, Minas Gerais Brazil; 4 Department of Uveitis, São Geraldo Hospital, Universidade Federal de Minas Gerais, Belo Horizonte, Brazil; 5 Department of Oral Biology, School of Dental Medicine, University at Buffalo, The State University of New York, Buffalo, USA; 6 Departament of Locomotor Apparatus, School of Medicine, Universidade Federal de Minas Gerais, Belo Horizonte, Brazil; 7 Hospital das Clínicas, Universidade Federal de Minas Gerais, Belo Horizonte, Minas Gerais Brazil

**Keywords:** Biomarkers, Cytokines, Saliva, Siglec-5, Sjögren’s disease, Non-Sjögren sicca

## Abstract

**Background:**

Sjögren disease (SjD) has a multifactorial pathogenesis that is not fully understood. Perceptions of disease severity shape healthcare-seeking behavior and engagement with diagnostic assessments, underscoring the need for objective biomarkers. Sialic acid-binding immunoglobulin-like lectins (siglecs) have emerged as relevant mediators in SjD immunopathology.

**Purpose:**

To investigate the diagnostic performance of siglec-5 expression in minor salivary gland (MSG) tissue and saliva samples from individuals with SjD and non-Sjögren *sicca* (nSS).

**Methods:**

A total of 109 participants with SjD and 41 with nSS were included. Salivary concentrations of siglec-5/siglec-14, inflammatory markers (IL-6, IL-8, IFN-γ, IgA, IgG, nitric oxide [NO]), and neutrophil extracellular traps (NETs) were measured. Immunohistochemical analyses of siglec-5, CD20, and CD3 were performed on MSG specimens. The data were analyzed descriptively and analytically.

**Results:**

Salivary levels of siglec-5/siglec-14, IgA, IgG, NO, and NETs were significantly higher in the SjD group compared to the nSS group. Elevated salivary levels of siglec-5/siglec-14, IL-6, and IgG were found among individuals with severe dryness scores. Immunohistochemical staining for siglec-5 was more pronounced in SjD samples and significantly associated with CD20 and CD3 positivity as well as the presence of xerophthalmia. Tissue infiltration by siglec-5 had greater diagnostic accuracy for SjD (area under the curve: 73.1% [95% confidence interval: 58.2–85]) than both salivary and ocular *sicca* tests.

**Conclusion:**

Siglec-5 expression was increased in individuals with SjD, supporting its involvement in disease pathogenesis as well as its potential usefulness as a biomarker. The availability of objective salivary and tissue markers may improve diagnostic pathways for SjD, thereby facilitating patient engagement.

**Supplementary Information:**

The online version contains supplementary material available at 10.1007/s00011-026-02188-8.

## Introduction

Sjögren disease (SjD) is a systemic autoimmune disorder characterized by lymphocytic infiltration and progressive dysfunction of the salivary and lacrimal glands, resulting in oral and ocular dryness [1, 2]. Its pathogenesis involves a complex interplay between genetic susceptibility and immune dysregulation primarily mediated by B and T lymphocytes, leading to the chronic inflammation of affected organs and tissues [3]. Despite considerable advances, the diagnosis of SjD is often delayed, particularly among individuals who test negative for anti-Ro antibodies, underscoring the need for reliable, noninvasive diagnostic biomarkers [1, 4].

Saliva is a valuable biological fluid for biomarker discovery owing to its complex molecular composition and noninvasive collection process. Several salivary biomarkers have shown potential in the diagnosis of SjD, including carbonic anhydrase VI, lipocalin-2, and sialic acid-binding immunoglobulin-like lectin-5 (siglec-5) [1, 5]. Siglec-5 is a member of the CD33-related siglec subfamily and increasingly recognized in a variety of pathological conditions. Structurally, this lectin is characterized by four extracellular immunoglobulin-like (Ig-like) domains and two intracellular tyrosine-based signaling motifs [6–8]. Siglec-5 is expressed in monocytes, neutrophils, macrophages [9], and lymphocytes [10, 11]. Through glycan recognition, siglecs mediate cell-cell interactions and modulate cellular activity in both the innate and adaptive immune systems [10]. A recent study reported elevated levels of siglec-5 in the saliva and serum of patients with SjD; however, its clinical relevance and mechanistic role in the disease have not yet been fully clarified [12].

The dysregulation of cytokines and chemokines plays a central role in the pathogenesis of SjD. As the disease progresses, imbalances in these mediators may exacerbate both local and systemic inflammatory responses [13, 14]. Neutrophil extracellular traps (NETs), which are composed of DNA-bound histones and granule-derived enzymes, further contribute to the amplification of inflammation in SjD and other rheumatic diseases [15–18]. Additionally, elevated levels of interferons (IFNs) and interleukins (e.g., IL-6 and IL-8) have been consistently associated with more severe clinical phenotypes in SjD [19, 20]. These cytokines not only reflect sustained immune activation but also constitute potential targets for therapeutic intervention and disease monitoring [14, 21]. However, the interactions between these inflammatory mediators and tissue-resident immune pathways remain poorly understood. In particular, crosstalk among cytokines, NETs, and siglec-expressing immune cells within the glandular microenvironment remains largely unexplored.

The aims of the present study were as follows: (i) to analyze the concentration of siglec-5 in minor salivary gland (MSG) tissue and saliva samples alongside a panel of inflammatory markers in individuals with SjD and non-Sjögren *sicca* (nSS); (ii) to investigate associations between these biomarkers and clinicopathological features of the diseases; and (iii) to assess the diagnostic performance of siglec-5 in classifying SjD using the criteria of the 2016 American College of Rheumatology and European League Against Rheumatism (ACR/EULAR) as the reference standard. We hypothesized that siglec-5 is involved in the pathogenesis of SjD, potentially contributing to disease progression through the recruitment of inflammatory cells and mediators.

## Materials and methods

### Study design and ethical aspects

An observational cross-sectional study was conducted. Data collection took place at the Departments of Rheumatology and Dentistry of the hospital affiliated with *Universidade Federal de Minas Gerais* in the city of Belo Horizonte, Brazil. Clinical data were obtained through an active review of medical records, with the concurrent collection of biospecimens. This study followed the Strengthening the Reporting of Observational Studies in Epidemiology guidelines [22] and received approval from the institutional research ethics committee (certificate no. 45125921400005149). All participants provided written informed consent.

### Study population

The SjD group comprised individuals with complaints of dry mouth and/or dry eye who met the 2016 ACR/EULAR classification criteria for SjD [4]. The control group consisted of individuals with xerostomia and/or xerophthalmia who underwent active investigation but did not meet these diagnostic criteria (nSS group). Participants were consecutively recruited between 2022 and 2024, and eligibility was confirmed by two clinicians (D.C.C. and L.A.T.). Based on ACR/EULAR guidelines, the exclusion criteria included a history of head and neck radiotherapy, polymerase chain reaction-positive hepatitis C infection, AIDS, sarcoidosis, amyloidosis, graft-versus-host disease, and IgG4-related disease.

### Clinical assessment

The variables of interest were demographic characteristics, disease duration, medication use, serological status for anti-Ro/SSA and anti-La/SSB antibodies, and ocular test results (Schirmer test and Ocular Staining Score). Symptoms of dryness (i.e., xerostomia/hyposalivation and xerophthalmia) were assessed in conjunction with the Clinical Oral Dryness Score [23].

### Saliva collection and analysis

Unstimulated saliva was collected using the spitting method over a 10-minute period, as described elsewhere [24]. All samples were obtained in the morning under fasting conditions. The participants were instructed to refrain from eating, toothbrushing, and smoking for at least one hour beforehand and to avoid speaking during the procedure. All collections were performed under the supervision of trained dentists. Saliva samples were aliquoted and stored at -80 °C until analysis. The salivary flow rate was expressed as ml/min. Inflammatory molecules in saliva were quantified using enzyme-linked immunosorbent assay (ELISA) kits: Human siglec-5/siglec-14 DuoSet (pg/ml), Human IL-6 Quantikine HS ELISA (pg/ml), Human IL-8/CXCL8 DuoSet (pg/ml), and Human IFN-γ DuoSet (R&D Systems^®^, Minneapolis, MN, USA; pg/ml); Human IgA (ng/ml) and Human IgG (ng/ml) ELISA kits (Wuhan Fine Biotech Co., Ltd., Wuhan, China); and the Nitrate/Nitrite Colorimetric Assay Kit (#780001, Cayman Chemical Co., Ann Arbor, MI, USA; µM). Quantification was performed using a standard curve specific to each marker generated with the corresponding recombinant cytokine. Salivary NETs were quantified following the methods described by Oliveira et al. [15]. An antibody (PA5-16672, dilution 1:500; Thermo Fisher Scientific Inc., Waltham, MA, USA) bound to a 96-well clear-bottom black plate captured the enzyme myeloperoxidase (MPO). The amount of DNA bound to the enzyme was quantified using the Quant-iT™ PicoGreen^®^ dsDNA Assay Kit (Thermo Fisher Scientific Inc., Waltham, MA, USA). Fluorescence intensity (excitation at 488 nm and emission at 525 nm) was measured with a FlexStation 3 Microplate Reader (Molecular Devices, San Jose, CA, USA). All assays were performed in duplicate, following the manufacturers’ protocols. Detailed information on sources, reagents, manufacturers, and software is provided in **Supplementary File 1**.

### MSG biopsy and histopathology

MSG biopsies were obtained from the lower lip of the participants. Histopathological analysis followed the criteria proposed by Fisher et al. [25], with the focus score defined as the number of lymphocytic foci per 4 mm^2^ of glandular tissue. Histological slides were independently reviewed by two oral pathologists (S.F.S. and T.A.S.), who examined serial sections while blinded to the clinical data. Discrepancies in interpretation were resolved by consensus.

### Immunohistochemistry

Serial sections approximately 5 µm in thickness were obtained with a microtome, mounted on polarized slides (Perfecta^®^, São Paulo, SP, Brazil), and subjected to immunohistochemical (IHC) analysis. Siglec-5 immunostaining was performed using a polyclonal anti-siglec-5 antibody (dilution 1:200; Thermo Fisher Scientific Inc., Waltham, MA, USA), with bone marrow tissue serving as the positive control [26] and phosphate-buffered saline as the negative control. Additional IHC markers included anti-CD20cy (ready-to-use monoclonal mouse anti-human CD20cy, clone L26, Agilent Dako^®^) and CD3 (ready-to-use polyclonal rabbit anti-human CD3, GA503, Agilent Dako^®^). The staining procedure was performed using the EnVision FLEX Mini Kit at high pH (Agilent Dako^®^, Santa Clara, CA, USA). Detection was performed with the 3,3’-diaminobenzidine chromogen included in the kit, followed by counterstaining with Mayer’s hematoxylin solution (Sigma Aldrich, St. Louis, MO, USA).

Immunostaining was quantified by a single examiner (F.L.A.L.C.) using an Axioskop 40 microscope (Zeiss, Goettingen, Germany) through manual counting of positive cells in all microscopic fields, each corresponding to an area of 0.1024 mm^2^ at 400× magnification. The analysis included the total number of positive cells, number of positive cells per mm^2^, and percentage of positive cells within the lymphocytic focal area. A second blinded examiner (V.Z.D.) performed counts on 10% of the slides and the intraclass correlation coefficient (ICC) was calculated, yielding a value above 0.85. Prior to quantification, two senior pathologists (T.A.S. and S.F.S.) jointly reviewed the slides with the examiners to ensure calibration and standardization of the assessment criteria. A diagram displaying the number of patients included in the study as well as those analyzed by IHC and salivary assays is provided in **Supplementary File 2**.

### Data analysis

The Statistical Package for the Social Sciences (SPSS) (version 25.0, Armonk, USA), GraphPad Prism (version 7.00, La Jolla, USA), Jamovi Software (The Jamovi Project, version 2.5), and R software (version 4.5.0; R Core Team, 2025) were used. Continuous variables were expressed as median (interquartile range) and categorical variables were expressed as absolute and relative frequencies. The Shapiro–Wilk test was used to assess normality. Group comparisons were performed using the Mann-Whitney *U* test or the Kruskal–Wallis test, with the Bonferroni correction applied for multiple comparisons. Categorical variables were analyzed using the chi-square test. Correlations between continuous variables were assessed using Spearman’s rank correlation coefficient. A significance level of *p* < 0.05 was adopted for all analyses. Receiver operating characteristic (ROC) curve analyses were performed using the MedCalc software (BVBA; Ostend, Flanders, Belgium). Sensitivity, specificity, and area under the curve (AUC), with corresponding 95% confidence intervals, were calculated for salivary and tissue siglec-5 and anti-Ro/SSA as well as the Schirmer test, Ocular Staining Score, and focus score. The Youden index was used to determine the optimal cutoff points for salivary and tissue siglec-5 as well as the focus score.

The statistical power analysis for non-parametric tests comparing the ELISA (saliva) and IHC (tissue) results for siglec-5 was performed assuming α = 0.05 (two-tailed). The required sample sizes to achieve 80% and 90% power were estimated by iteratively increasing the *N* until the desired power thresholds were reached for the observed effect sizes. The calculations were conducted in Python 3.11 (SciPy, NumPy, Pandas) within the Google Colab environment (Google LLC, Mountain View, CA, USA).

## Results

### Clinicodemographic data

This study included 150 participants: 109 with SjD (103 women, six men; age range: 10–76 years) and 41 with nSS (36 women, five men; age range: 14–79 years). Mean symptom duration was 138 months (range: 12–616 months) in both groups. In the SjD group, the mean time to diagnosis (diagnostic delay) was 86.5 months (range: 12–432 months). Xerophthalmia was significantly more frequent in the SjD group (*p* = 0.021). Higher positivity rates for the Schirmer test and Ocular Staining Score were found in the SjD group (*p* = 0.021 and *p* = 0.002, respectively). Although the frequency of xerostomia did not differ between groups, the individuals with SjD had significantly lower unstimulated whole salivary flow rates (*p* = 0.003) and a higher frequency of hyposalivation (*p* = 0.026) (Table 1). Among the patients in the SjD group, 69.7% were anti-Ro/SSA positive and 86.2% had a focus score of ≥ 1 (data not shown).

**Table 1:** Clinicodemographic characteristics of the patients included in the study

Variables	SjD (*n* = 109)	nSS (*n* = 41)	*p* value
Age in years, median (range)	58 (10–76)	57.5 (14–79)	0.543^§^
*Sex, n (%)*			
Female	103 (94.5)	36 (87.8)	0.173^ǂ^
Male	6 (5.5)	5 (12.2)	
*Tobacco smoking, n (%)*			
Never	76 (69.7)	30 (73.2)	0.247^¶^
Still	2 (1.8)	1 (2.4)	
Stopped	13 (11.9)	2 (4.9)	
NR	18 (16.5)	8 (19.5)	
*Alcohol consumption, n (%)*			
Never	81 (74.3)	31 (75.6)	1.000^¶^
Still	9 (8.4)	1 (2.4)	
Stopped	5 (4.7)	3 (7.3)	
NR	14 (12.8)	6 (14.7)	
*Xerostomia, n (%)*			
Yes	87 (79.8)	35 (85.4)	
No	16 (14.7)	5 (12.2)	
NR	6 (5.5)	1 (2.4)	
*Xerophthalmia, n (%)*			
Yes	80 (73.4)	23 (56.1)	
No	15 (13.8)	12 (29.3)	
NR	14 (12.8)	6 (14.6)	
*Schirmer test, n (%)*			
Positive	25 (22.9)	2 (4.9)	**0.021** ^**ǂ**^
Negative	35 (32.1)	14 (34.2)	
NR	49 (44.9)	25 (60.9)	
*Ocular Staining Score, n (%)*			
Positive	33 (30.3)	2 (4.9)	**0.002** ^**ǂ**^
Negative	24 (22.0)	14 (34.2)	
NR	52 (47.7)	25 (60.9)	
*Anti-Ro/SSA, n (%)*			
Positive	76 (69.7)	5 (12.2)	
Negative	27 (24.8)	36 (87.8)	
NR	6 (5.5)	0	
Focus score, median (range)	2.0 (0–7.7)	0.4 (0–6.3)	**< 0.001** ^**§**^
Unstimulated whole salivary flow rate (mL/min), median (range)	0.10 (0–0.91)	0.18 (0.02–0.8)	**0.003** ^**§**^
*Hyposalivation, n (%)*			
Yes	77 (70.6)	21 (51.2)	
No	32 (29.4)	20 (48.8)	
*Parotitis, n (%)*			
Yes	45 (41.3)	11 (26.8)	
No	55 (50.5)	25 (61)	
NR	9 (8.3)	5 (12.2)	

Values in bold indicate statistical significance (p < 0.05)

NR, not reported; nSS, non-Sjögren sicca; SjD, Sjögren disease.

^§^Mann-Whitney test.

^‡^Fisher exact test.

Linear-by-linear association.

^ǂ^

**Supplementary Files 3** and **4** present comparative analyses of comorbidities and medication use. The SjD group had a greater frequency of thyroid diseases than the nSS group (*p* = 0.034) as well as more frequent use of thyroxine (*p* = 0.033) and vitamin D (*p* = 0.004). A total of 21.9% of the patients in the nSS group had concomitant autoimmune diseases, including rheumatoid arthritis and systemic lupus erythematosus. Patients with isolated SjD were compared to those with SjD overlapping other rheumatic diseases. Anti-Ro/SSA antibody positivity was more frequent among those with overlapping SjD, whereas no significant differences between subgroups were found for demographic characteristics, dryness measures, or the focus score **(Supplementary File 5)**.

### Siglec-5 expression in MSG and saliva

**Fig. 1:**
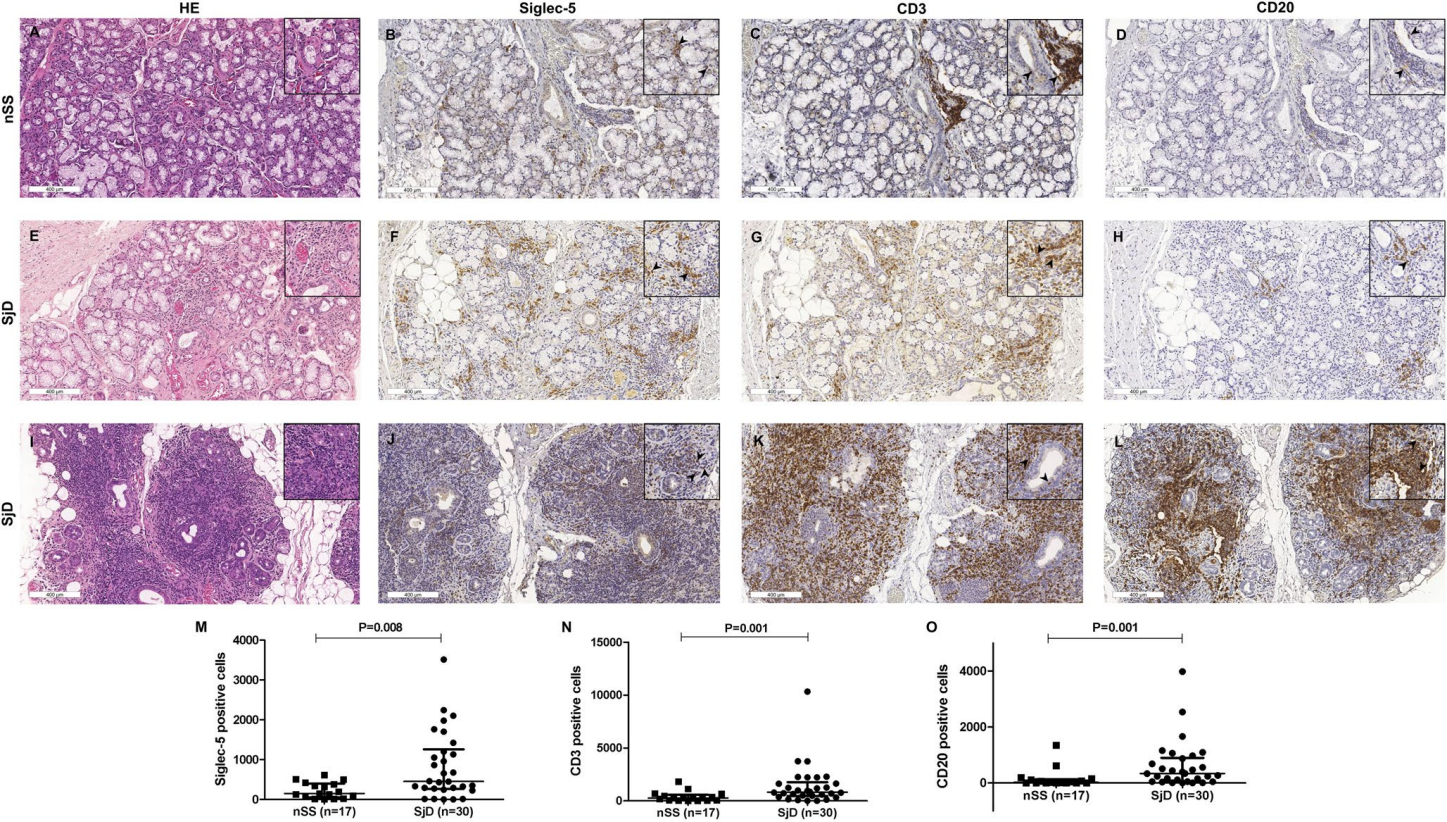
Siglec-5, CD3, and CD20 expression in minor salivary glands. **(A, E**,** I)** Representative hematoxylin and eosin-stained sections and immunohistochemical staining for **(B**,** F**,** J)** siglec-5, **(C**,** G**,** K)** CD3, and **(D**,** H**,** L)** CD20 in minor salivary glands from individuals with non-Sjögren *sicca* (nSS) and Sjögren disease (SjD). Quantitative analysis revealed greater expression of **(M)** siglec-5, **(N)** CD3, and **(O)** CD20 in SjD compared to nSS. Arrows identify positive cells. Each dot represents one patient. Horizontal lines indicate median and interquartile range. Statistical comparisons performed using the Mann-Whitney *U* test

**Fig. 2:**
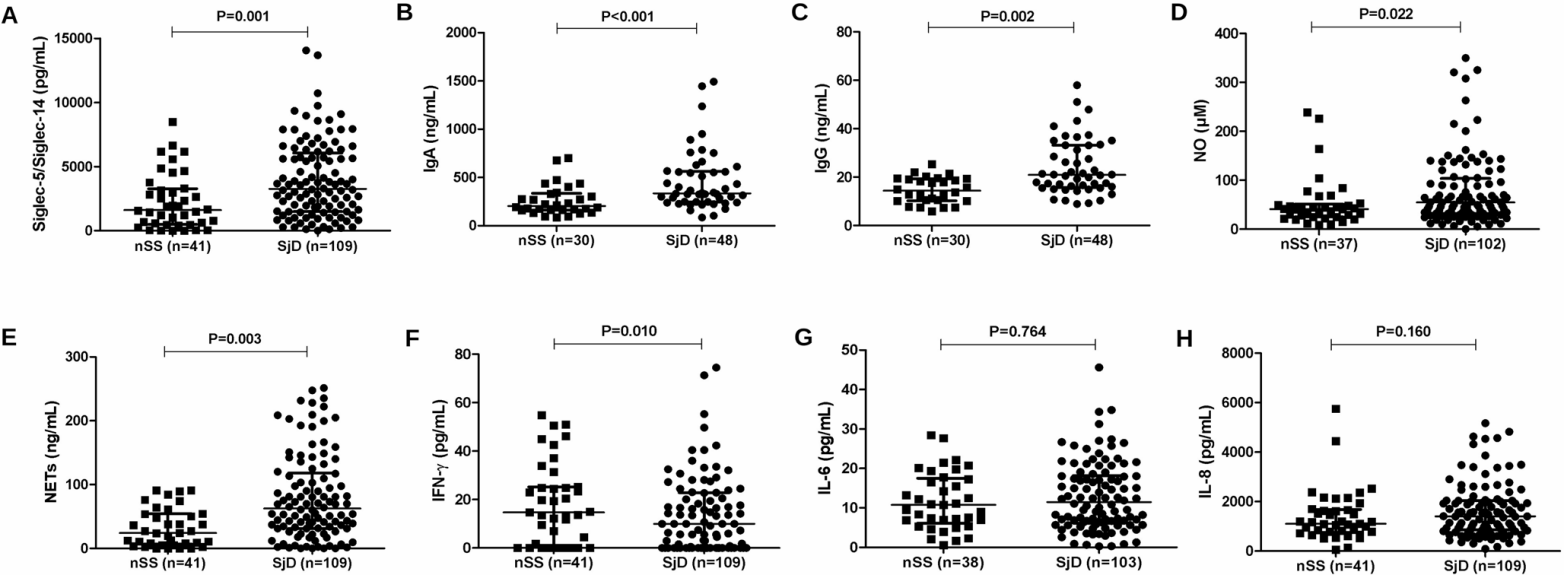
Salivary inflammatory markers in Sjögren disease (SjD) and non-Sjögren *sicca* (nSS). Salivary concentrations of **(A)** siglec-5/siglec-14, **(B)** IgA, **(C)** IgG, **(D)** nitric oxide (NO), **(E)** neutrophil extracellular traps (NETs), **(F)** interferon gamma (IFN-γ), **(G)** interleukin (IL)-6, and **(H)** IL-8 in individuals with SjD and nSS. Statistical comparisons performed using the Mann–Whitney *U* test. Each dot represents one patient. Horizontal lines indicate median and interquartile range

Siglec-5 expression was consistently higher in the SjD group in both tissue and salivary samples and was associated with inflammatory cell infiltration. IHC analysis confirmed stronger siglec-5 staining in SjD samples (*p* = 0.008), predominantly on mononuclear inflammatory cells and occasionally on neutrophils. CD3 and CD20 immunostaining was also higher in the SjD group compared to the nSS group (*p* = 0.001 for both markers), confirming the presence of T- and B-cell infiltrates, respectively (Fig. 1). Siglec-5-positive cells were more evenly distributed throughout glandular tissue (mean: 17.8% per focus), whereas CD3- and CD20-positive cells were predominantly concentrated within inflammatory foci (mean: 53.2% and 58.1%, respectively). The distribution patterns are illustrated in **Supplementary File 6**. Salivary levels of siglec-5 were significantly higher in the SjD group (*p =* 0.001) (Fig. 2A).

### Salivary inflammatory markers

SjD was characterized by a distinct salivary inflammatory profile marked by increased immunoglobulins, oxidative mediators, and neutrophil-derived products. Individuals with SjD had significantly higher salivary levels of IgA (*p* < 0.001), IgG (*p* = 0.002), NO (*p* = 0.022), and NETs (*p* = 0.003) compared to the nSS group (Fig. 2B–E). In contrast, IFN-γ levels were significantly higher in the nSS group (*p* = 0.010) (Fig. 2F). No significant differences between groups were found for IL-8 or IL-6 (Fig. 2G–H). No differences in salivary cytokines or immunohistochemical markers were detected when comparing individuals with isolated SjD and those with SjD overlapping other rheumatological diseases (data not shown).

### Correlation between clinical parameters, salivary siglec-5/siglec-14, and inflammatory markers

Moderate correlations were found between salivary siglec-5/siglec-14 and IL-8, IgA, and NETs in the nSS group **(Supplementary File 7 A)**. Salivary siglec-5/siglec-14 was moderately correlated with IL-8, IL-6, IgG, IFN-γ, and NETs in the SjD group **(Supplementary File 7B)**. Strong positive correlations were identified between IgA and IgG as well as between CD20 and CD3.

### Association between dryness and inflammatory markers in MSG and saliva

Clinical measures of dryness were associated with increased inflammatory activity in both salivary gland tissue and saliva. Individuals with severe oral dryness, as assessed by the Clinical Oral Dryness Score, had higher focus scores (*p* = 0.042) as well as higher salivary levels of siglec-5/siglec-14 (*p* = 0.027), IL-6 (*p* = 0.022), and IgG compared to those with moderate dryness (*p* = 0.031). Patients with moderate dryness had significantly higher NET levels than those with mild dryness (*p* = 0.031) (Table 2). Xerophthalmia was associated with increased inflammatory activity in salivary gland tissue, whereas only IgG levels were significantly elevated in saliva (Table 3). Xerostomia was associated with higher salivary levels of IL-6, NO, and NETs (*p* < 0.05) **(Supplementary File 8)**.

**Table 2:** Association between quantitative variables and the clinical oral dryness score

Variables^‡^	Mild dryness (0–3)		Moderate dryness (4–6)		Severe dryness (7–10)		*p* value^§^
Unstimulated whole salivary flow rate	*n =* 48	0.1 (0–0.8) ^A^	*n =* 91	0.1 (0–0.9) ^A^	*n =* 11	0.1 (0–0.7) ^A^	0.154
Focus score	*n =* 46	1.3 (0–5.4) ^A^	*n =* 78	1.3 (0–7.7) ^AB^	*n =* 10	1 (0–3.9) ^B^	**0.047**
Siglec-5 total positive cells	*n =* 13	147 (3.0–3,511) ^A^	*n =* 28	440.5 (0–2,240) ^A^	*n =* 6	365 (6–1,203) ^A^	0.108
Siglec-5 total positive cells per foci	*n =* 13	15 (0–815) ^A^	*n =* 28	74 (0–568) ^A^	*n =* 6	102.5 (0–317) ^A^	0.343
CD20 total positive cells	*n =* 13	74 (0–1,158) ^A^	*n =* 28	134.5 (0–3,948) ^A^	*n =* 6	560 (0–1,092) ^A^	0.475
CD20 total positive cells per foci	*n =* 13	45 (0–1,032) ^A^	*n =* 28	50.5 (0–3,669) ^A^	*n =* 6	503 (0–1,001) ^A^	0.418
CD3 total positive cells	*n =* 13	472 (0–2,286) ^A^	*n =* 28	576.5 (9–10,334) ^A^	*n =* 6	850 (49–2,260) ^A^	0.605
CD3 total positive cells per foci	*n =* 13	203 (0–2,005) ^A^	*n =* 28	232.5 (0–9,739) ^A^	*n =* 6	693 (0–2,006) ^A^	0.349
Saliva Siglec-5/Siglec-14 (pg/mL)	*n =* 48	1,960.5 (1.7–8,971.9) ^A^	*n =* 91	3,149.1 (0–17,642) ^AB^	*n =* 11	5,051.2 (935.9–10,724) ^B^	**0.027**
Saliva IL-8 (pg/mL)	*n =* 48	1,041.1 (87–4,535.2) ^A^	*n =* 91	1,546.4 (56.3–13,424.1) ^A^	*n =* 11	1,412.4 (955.5–4,573.1) ^A^	0.051
Saliva IL-6 (pg/mL)	*n =* 46	8.6 (0.2–31.2) ^A^	*n =* 85	15.3 (0.6–146.7) ^AB^	*n =* 10	21 (3.9–26.6) ^B^	**0.022**
Saliva IgA (ng/mL)	*n =* 29	315.2 (85.6–759.4) ^A^	*n =* 43	275.9 (85.5–1,492) ^A^	*n =* 6	392.1 (179.8–754.9) ^A^	0.430
Saliva IgG (ng/mL)	*n =* 29	19.2 (5.8–57.9) ^AB^	*n =* 46	17.5 (7.3–64.8) ^A^	*n =* 6	34.8 (19.6–47.9) ^B^	**0.031**
Saliva IFN-γ (pg/mL)	*n =* 48	9.7 (0–50.5) ^A^	*n =* 91	7.4 (0–180.1) ^A^	*n =* 11	13.4 (0–40.4) ^A^	0.725
Saliva NO (µM)	*n =* 44	41.4 (5.3–595.2) ^A^	*n =* 86	47.3 (0–349.6) ^A^	*n =* 9	58.7 (15.1–225.9) ^A^	0.733
Saliva NETs (ng/mL)	*n =* 48	35.9 (0–398.1) ^A^	*n =* 91	60.8 (0–1,359) ^B^	*n =* 11	64.1 (3.8–204.7) ^AB^	**0.031**

Values in bold indicate statistical significance (p < 0.05)

IgA, immunoglobulin A; IgG, immunoglobulin G; IL, interleukin; IFN, interferon; NETs, neutrophil extracellular traps; NO, nitric oxide; Siglec, sialic acid-binding immunoglobulin-like lectin.

^‡^Median and range.

*p* values were obtained using the Kruskal–Wallis test. Data are expressed as median (range).^A,^^B^Superscript letters indicate pairwise comparisons: identical letters denote no significant difference, while different letters denote a statistically significant difference.

^§^All pairwise comparisons were conducted using Bonferroni correction methods.

**Table 3:** Association between xerophthalmia and immune marker positivity (siglec-5, CD20, and CD3), as well as cytokine expression in salivary gland tissue and saliva

Variables^‡^		Xerophthalmia		
Tissue		Positive (*n* = 27)	Negative (*n* = 9)	*p* value
Total siglec-5 positive cells		453 (0–3,511)	109.5 (10–616)	0.117^§^
Total siglec-5 positive cells per foci		86.5 (0–815)	0 (0–347)	**0.009** ^**§**^
Total CD20 positive cells		217.5 (0–3,984)	25.5 (0–1,354)	**0.029** ^**§**^
Total CD20 positive cells per foci		172.5 (0–3,669)	3.5 (0–1,354)	**0.021** ^**§**^
Total CD3 positive cells		819.5 (13–10,334)	173 (0–1,830)	**0.003** ^**§**^
Total CD3 positive cells per foci		566 (0–9,739)	16 (0–1,495)	**0.005** ^**§**^

Saliva		Positive	Negative	
Siglec-5/Siglec-14, pg/mL (*n* = 103 positive; *n* = 27 negative)		1,345.1 (0–6,654.7)	1,383.1 (1.7–5,718.3)	0.673^§^
IL-8, pg/mL (*n* = 103 positive; *n* = 27 negative)		943.1 (56.3–2,669.5)	886.6 (623.7–2,152.2)	0.951^§^
IL-6, pg/mL (*n* = 97 positive; *n* = 25 negative)		7.3 (0.2–25.7)	12.8 (3.2–17.2)	0.433^§^
IgA, ng/mL (*n* = 53 positive; *n* = 13 negative)		327.7 (85.5–950.4)	258.1 (85.6–475.2)	0.076^§^
IgG, ng/mL (*n* = 53 positive; *n* = 13 negative)		21.1 (7.3–64.8)	14.8 (10.5–20.3)	**0.010** ^**§**^
IFN-γ, pg/mL (*n* = 103 positive; *n* = 27 negative)		22.8 (0–110.5)	13.5 (0–50.9)	0.649^§^
NO, µM (*n* = 97 positive; *n* = 23 negative)		38.6 (8.7–325.1)	46.9 (21.6–238.6)	0.957^§^
NETs, ng/mL (*n* = 103 positive; *n* = 27 negative)		25.1 (0–204.7)	26.9 (2.6–103.3)	0.202^§^

Values in bold indicate statistical significance (p < 0.05)

IgA, immunoglobulin A; IgG, immunoglobulin G; IL, interleukin; IFN, interferon; NETs, neutrophil extracellular traps; NO, nitric oxide; Siglec, sialic acid-binding immunoglobulin-like lectin.

^§^Mann-Whitney test.

### Diagnostic potential of siglec-5

**Fig. 3:**
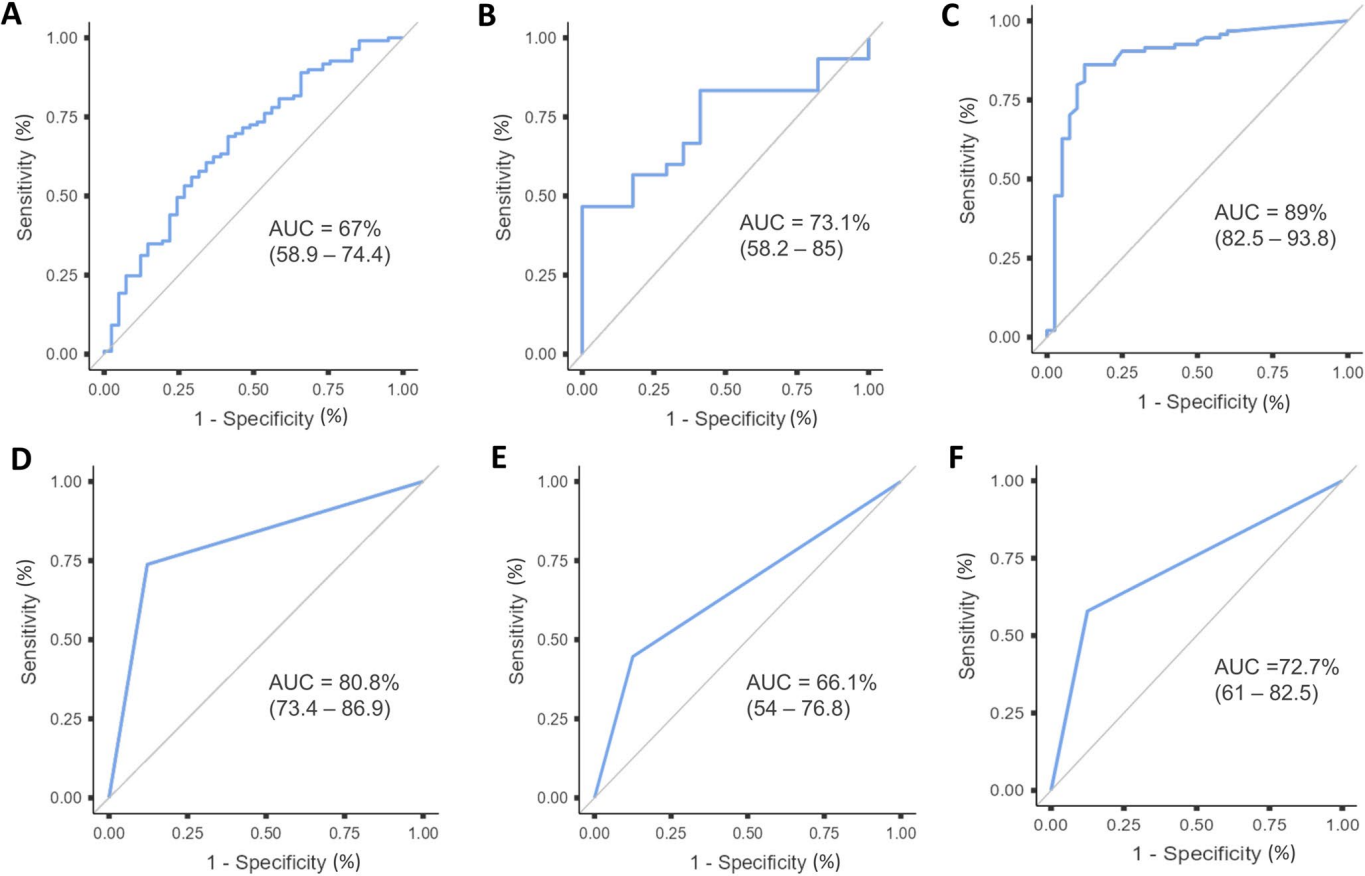
Diagnostic performance of siglec-5 and established markers of Sjögren disease. Area under the curve (AUC) for **(A)** salivary siglec-5, **(B)** tissue siglec-5, expressed as the total number of positive cells, **(C)** focus score, **(D)** anti-Ro/SSA antibodies, **(E)** Schirmer test, and **(F)** Ocular Staining Score

Tissue siglec-5 demonstrated moderate diagnostic accuracy for SjD, outperforming several functional *sicca* tests, but remaining inferior to established classification markers. Tissue siglec-5 exhibited greater diagnostic accuracy (AUC: 73.1% [95% CI: 58.2–85]) than salivary siglec-5/siglec-14 (AUC: 67% [95% CI: 58.9–74.4]), the Schirmer test (AUC: 66.1% [95% CI: 54–76.8]), and the Ocular Staining Score (AUC: 72.7% [95% CI: 61–82.5]). Anti-Ro/SSA antibodies and the focus score outperformed all other parameters (AUC: 80.8% [95% CI: 73.4–86.9] and AUC: 89% [95% CI: 82.5–93.8], respectively) (Fig. 3). Sensitivity, specificity, the positive predictive value, negative predictive value, Youden index, and optimal cutoff values are given in **Supplementary File 9**. Comparisons between AUCs using DeLong’s test are shown in **Supplementary File 10.** Combined ROC curves are presented in **Supplementary File 11**.

### Post hoc power analysis

The *post hoc* power analysis indicated that both saliva- and tissue-based comparisons achieved an approximately 70% power (1–β ≈ 0.70; i.e., moderate) with a two-tailed α of 0.05.

## Discussion

This study demonstrated that siglec-5 is significantly upregulated in both saliva and MSG samples from individuals with SjD compared to those with nSS. Its expression was predominantly observed in mononuclear inflammatory cells and was associated with elevated salivary concentrations of IgA, IgG, NO, and NETs. Higher salivary levels of siglec-5/siglec-14 and IL-6 were also associated with greater clinical dryness severity. Tissue siglec-5 demonstrated superior diagnostic performance compared to salivary and ocular tests, with 100% specificity, although overall accuracy remained lower than that of anti-Ro/SSA antibodies and the focus score. These findings support the hypothesis that siglec-5 is involved in the pathogenesis of SjD, potentially contributing to the exacerbation of glandular inflammation. Given that MSG biopsy is routinely used in the diagnostic workup of SjD, it may be plausible to incorporate the assessment of siglec-5 alongside the focus score to improve diagnostic precision.

Lee et al. [12] were the first to report elevated salivary levels of siglec-5 in individuals with SjD using the ACR/EULAR 2002/2012 classification criteria and focusing predominantly on salivary fluid. The present study advances this evidence by exclusively applying the updated ACR/EULAR 2016 criteria and incorporating the IHC analysis of glandular tissue. The robust detection of siglec-5 within periductal inflammatory foci (predominantly on mononuclear cells) is consistent with its known expression on myeloid cells and B lymphocytes [10]. The observed co-expression of siglec-5 and CD20 in MSG tissue further implicates B cells in the local upregulation of this lectin, reinforcing prior findings suggesting that siglec-5 may regulate humoral immunity by modulating B-cell activation thresholds [27].

Siglecs regulate immune responses through immunoreceptor tyrosine-based inhibitory motif (ITIM)-mediated signaling. Siglec-5, in particular, is expressed in myeloid cells and B lymphocytes, modulating their activation thresholds [10]. Although earlier studies suggested limited expression in T cells [28], more recent evidence indicates that activated T cells can express siglec-5 through post-translational mechanisms [11]. This raises important questions regarding its functional role in the lymphocytic infiltrates characteristic of SjD. It remains unclear whether siglec-5 acts as a regulatory brake—favoring B-cell hyperactivation and subsequent accumulation in glandular tissue—or contributes to inflammatory amplification through aberrant signaling that modulates T-cell responses. The crosstalk between salivary glands and B and T lymphocytes promotes the expansion of autoantibody-producing plasma cells and the release of pro-inflammatory cytokines, reinforcing their pathogenic role in glandular dysfunction [19]. Our findings of positive correlations between salivary siglec-5/siglec-14 and IL-8, IL-6, IgG, IFN-γ, and NETs lend strength to the latter hypothesis.

As previously reported [12], the ELISA kit used in this study detects both siglec-5 and 14. These two proteins have extensive similarity in their amino-terminal regions. Yamanaka et al. [29] showed that siglec-14 is expressed in granulocytes and monocytes, while siglec-5 is predominantly expressed in B cells. The authors also found that some individuals lack siglec-14 expression while retaining intact siglec-5 expression, which is a phenomenon hypothesized to result from a genetic event that fuses segments of *SIGLEC14* and *SIGLEC5*, generating a novel fusion gene denominated *SIGLEC14/5*. Therefore, it is likely that the predominant protein measured in saliva corresponds to siglec-5. However, we acknowledge that salivary quantification is not specific to siglec-5 and may be influenced by *SIGLEC14/5* polymorphisms. Future studies using protein-analytical techniques are warranted to enable precise discrimination between these isoforms.

Aside from siglec-5, the present study identified a distinct salivary inflammatory signature in SjD characterized by elevated levels of NO, IgA, IgG, and NETs. These molecules may serve as potential salivary biomarkers of disease activity and immune dysregulation in SjD [19, 30, 31]. Although IL-6 levels did not differ between the two subgroups, this cytokine was associated with symptom severity, as measured by the Clinical Oral Dryness Score. Increased salivary IgA and IgG levels likely reflect local plasma cell activation and may contribute to glandular injury through immune complex formation and complement activation [32]. The correlations with the focus score and clinical dryness indices demonstrate their diagnostic importance and are consistent with previous findings linking immunoglobulin levels to tissue damage in SjD [33, 34]. It is noteworthy that no differences were found in salivary cytokines when comparing individuals with isolated SjD and those with SjD overlapping other rheumatological diseases, thus raising the possibility that the presence of an additional autoimmune disease may not constitute a distinct immunological phenotype [2].

The involvement of NETs in the pathogenesis of SjD has gained increasing recognition, particularly following the study by Peng et al. [16], who demonstrated NET accumulation in plasma and labial salivary glands in affected individuals and correlated with disease activity. Although neutrophils are not traditionally regarded as central players in the histopathology of SjD, their ability to infiltrate periductal regions and contribute to local tissue damage—particularly during early or active disease stages—has been documented [35, 36]. The present study advances this field by reporting, for the first time, elevated levels of NETs in saliva, lending strength to the notion that salivary content reflects dynamic inflammatory processes occurring on the glandular level. Importantly, NET formation is driven by oxidative stress, which is a mechanism long implicated in the immunopathogenesis of SjD [37]. Thus, both NET induction and NO overproduction may synergically contribute to epithelial injury [38, 39].

The mechanistic interplay between NETs and siglec-5 also warrants consideration. Neutrophils express siglec-5 on their surface and inflammatory conditions can induce the proteolytic shedding of siglecs into soluble forms that are detectable in extracellular fluids [40, 41]. The concurrent elevation of both NETs and siglec-5 in the saliva of individuals with SjD may therefore reflect a shared cellular origin, suggesting that activated or dysregulated neutrophils contribute to both phenomena. Furthermore, siglec-5 contains an ITIM that would be expected to attenuate cellular activation under homeostatic conditions [42]. In autoimmunity, however, the balance between activating and inhibitory signals may become disrupted [43]. Taken together, these observations position siglec-5 at the interface between innate and adaptive immune responses in SjD, linking neutrophil-driven NETosis, oxidative stress, and B cell–mediated autoimmunity in the glandular microenvironment. The possibility that siglec-5 upregulation constitutes a failed inhibitory feedback mechanism rather than a protective signal merits further investigation and may offer insight into immune-checkpoint dysfunctions in cases of chronic exocrinopathy [44].

This study is the first to demonstrate siglec-5 expression in inflammatory cells in a Brazilian cohort. It was conducted at a tertiary care center with the involvement of experienced healthcare providers, including dentists, rheumatologists, and ophthalmologists, enabling a comprehensive clinical and laboratory assessment. The study also has limitations that should be acknowledged. First, some of the participants were undergoing pharmacological treatment, which may have influenced the expression of inflammatory markers. Second, both SjD and nSS are clinicopathologically heterogeneous conditions and this variability may have contributed to divergent cytokine profiles. Therefore, it is important to note that the comparison group (i.e., patients with nSS) had concomitant immune-mediated rheumatic conditions, which may have affected the discriminative performance of siglec-5 between SjD and nSS. Third, the relatively small sample size and the cross-sectional design limit the generalizability of the findings. Although this study attempted to correlate cytokine levels with dryness indices, these clinical scores may not fully capture the multifactorial nature of xerostomia, as this symptom is self-reported and may introduce reporting bias and subjective variability. The power analysis was based solely on the observed effect sizes and the available sample. Prospective cohort studies including healthy controls are needed to enable a more in-depth exploration of biological clinical parameters. Lastly, SjD is a multifactorial condition in which molecular, genetic, hormonal, and environmental mechanisms interact, ultimately leading to a substantial reduction in quality of life [45]. Elucidating the molecular pathways underlying the disease strengthens the comprehensive diagnosis and integrative healthcare. In this scenario, lifestyle interventions, such as regular physical activity and adherence to a balanced diet, may constitute adjunctive strategies to alleviate disease-related symptoms [46].

## Conclusion

Siglec-5 was upregulated in both saliva and MSG samples from individuals with SjD, correlating with markers of lymphocytic infiltration, clinical dryness, and other inflammatory mediators (IL-6, NO, and NETs). Siglec-5 also demonstrated good diagnostic accuracy in distinguishing SjD from nSS. These findings support the involvement of this lectin in the pathogenesis of SjD and highlight its diagnostic potential, particularly when measured in salivary gland tissue. Strategies blocking siglec-5 may be useful for the treatment of SjD, which is in line with ongoing clinical trials investigating anti-siglec antibodies in other autoimmune diseases [47]. Tissue expression of siglec-5 may serve as a complementary marker to current classification criteria, especially in seronegative patients or those with a borderline focus score, in whom biopsy is crucial and diagnostic confirmation is often more challenging [48]. Lastly, attention should also be given to patients with nSS, who experience significant symptoms despite lacking formal diagnostic criteria, underscoring the need for earlier recognition and more specific biomarkers of exocrinopathy.

## Supplementary Information

Below is the link to the electronic supplementary material.

Supplementary Material 1

## Data Availability

All data are available within the article or supplementary materials.
