# Supplementary material for: Diagnostic significance of salivary and glandular siglec-5 in Sjögren disease and non-Sjögren sicca
Source: Inflamm Res. 2026 Feb 26;75(1):42. doi: 10.1007/s00011-026-02188-8 (PMC12935824; doi:10.1007/s00011-026-02188-8)
Supplement: Supplementary file 1 — Supplementary Material 1 [file 11_2026_2188_MOESM1_ESM.docx]

**Supplementary Files**

**Diagnostic significance of salivary and glandular siglec-5 in Sjögren disease and non-Sjögren *sicca***

**Table of contents**

**Supplementary File 1 –** Antibodies, assay kits, sources, reagents, manufacturers, software and Research Resource Identifiers (RRIDs).......................................................1

**Supplementary File 2 -** Number of patients included in the study per assay...................6

**Supplementary File 3 -** Comparative analysis of comorbidity prevalence in patients with SjD and nSS.......................................................................................................................7

**Supplementary File 4 -** Comparative analysis of medication use in patients with SjD and nSS.....................................................................................................................................9

**Supplementary File 5 -** Clinicodemographic data of SjD and overlapping SjD..............11

**Supplementary File 6 -** Expression of inflammatory cells in SjD and nSS.....................12

**Supplementary File 7 -** Spearman correlation matrix of clinical parameters, salivary siglec-5 and inflammatory markers..................................................................................13

**Supplementary File 8 -** Association of xerostomia with immune marker positivity (siglec-5, CD20 and CD3) and cytokine expression in salivary gland..............................14

**Supplementary File 9 -** Evaluation of area under the curve (AUC), sensitivity, specificity, Youden index, and cut-off point of EULAR (SjD and nSS) in relation to measures..........................................................................................................................15

**Supplementary File 10 –** Pairwise AUC comparisons (DeLong’s test)..........................17

**Supplementary File 11 -** Combined ROC curves...........................................................19

**Supplementary File 1.** Antibodies, assay kits, sources, reagents, manufacturers, software and Research Resource Identifiers (RRIDs)

| Antibodies | | | | | | | | |
| --- | --- | --- | --- | --- | --- | --- | --- | --- |
| Target | **Application** | **Host** | **Reactivity** | **Clone/Cat No.** | **Dilution** | **Source** | **Lot No.** | **RRIDs** |
| Siglec-5 Polyclonal Antibody | IHC | Rabbit/ IgG | Human | Polyclonal PA5-11675 | 1:200 | ThermoFisher Scientific Inc. Waltham, MA, USA | YL4143926A | AB_2189281 |
| Polyclonal Rabbit Anti-Human CD3 | IHC | Rabbit | Human | Polyclonal GA503 | Ready-to-use | Agilent Dako Santa Clara, CA, USA | 41546367 | AB_3665673 |
| Monoclonal Mouse Anti-Human CD20cy | IHC | Mouse | Human | Monoclonal Clone L26 GA604 | Ready-to-use | Agilent Dako Santa Clara, CA, USA | 41614232 | AB_3678473 |
| Human Siglec-5/ Siglec-14 DuoSet | ELISA | - | Human | DY1072 | - | R&D Systems Minneapolis, MN, USA | P325298 | AB_3720330 |
| Human IgA (Immunoglobulin A) | ELISA | - | Human | EH0415 | - | Wuhan Fine Biotech Co. Wuhan, Hubei, China | H0415I045 | AB_3720331 |
| Human IgG (Immunoglobulin G) | ELISA | - | Human | EH0417 | - | Wuhan Fine Biotech Co. Wuhan, Hubei, China | H0417I045 | AB_3720332 |
| Human IFN-gamma DuoSet | ELISA | - | Human | DY285B-05 | - | R&D Systems Minneapolis, MN, USA | P364279 | AB_2928044 |
| Human IL-6 Quantikine | ELISA | - | Human | HS600C | - | R&D Systems Minneapolis, MN, USA | P341391 | AB_2893335 |
| Human IL-8/CXCL8 DuoSet | ELISA | - | Human | DY208-05 | - | R&D Systems Minneapolis, MN, USA | P362550 | AB_2892143 |
| Myeloperoxidase Polyclonal Antibody | ELISA | Rabbit/ IgG | Human, Mouse, Rat | PA5-16672 | 1:500 | ThermoFisher Scientific Inc. Waltham, MA, USA | - | AB_11006367 |
| Colorimetric Assay Kits | | | | | | | | |
| Name | **Application** | | | **Cat No.** | | **Source** | **Lot No.** | **RRIDs** |
| Nitrate/Nitrite | Biochemical colorimetric assay (Griess reaction-based) | | | 780014 | | Cayman Chemical Co. Ann Arbor, MI, USA | - | - |
| Quant-iT^TM^ PicoGreen^®^ dsDNA Assay Kit | Fluorescent quantification of dsDNA | | | P7589 | | ThermoFisher Scientific Inc. Waltham, MA, USA | 2247972 | - |
| Other supplies | | | | | | | | |
| Material | **Application** | | | **Cat No.** | | **Source** | | **RRIDs** |
| EnVision FLEX, Mini Kit, High pH (Link) | IHC | | | K8023 | | Agilent Dako, Santa Clara, CA, USA | | AB_2890017 |
| Polarized slides Perfecta | IHC | | | 301.615.810 | | Perfecta, São Paulo, SP, Brazil | | - |
| Antibody diluent (FLEX) | IHC | | | K8006 | | Agilent Dako, Santa Clara, CA, USA | | - |
| Citric Acid, Anhydrous | IHC | | | 01A1026.01.AG | | Synth, Diadema, SP, Brazil | | - |
| Mayer’s hematoxylin solution | IHC | | | MHS16 | | Sigma Aldrich, St. Louis, MO, USA | | - |
| Axioskop 40 Microscope | IHC | | | D-37030 | | Zeiss, Goettingen, Germany | | - |
| FlexStation 3 Multi-Mode Microplate Reader | ELISA | | | - | | Molecular Devices, San Jose, CA, USA | | SCR_025281 |
| BioTek Microplate Washer | ELISA | | | - | | BioTek Instruments (Agilent), Santa Clara, CA, USA | | SCR_019725 |
| Software | | | | | | | | |
| Resource | **Version** | | | **Manufacturer** | | **Location** | | **RRIDs** |
| IBM SPSS Statistics | 25.0 | | | IBM Corporation | | Armonk, NY, USA | | SCR_002865 |
| GraphPad Prism | 7.0 | | | GraphPad Software Inc. | | La Jolla, CA, USA | | SCR_002798 |
| Jamovi | 2.5 | | | The jamovi project | | Sydney, Australia | | SCR_016142 |
| R | 4.5.0 | | | R Core Team | | Vienna, Austria | | SCR_001905 |
| MedCalc | 23.3.2 | | | MedCalc Software Ltd. (BVBA) | | Ostend, Flanders, Belgium | | SCR_015044 |
| Python | 3.11 | | | Python Software Foundation | | Wilmington, DE, USA | | SCR_008394 |

**Note:** ELISA, Enzyme-linked immunosorbent assay; IgA, immunoglobulin A; IgG, immunoglobulin G; IHC, immunohistochemistry; IL, interleukin; IFN, interferon; NETs, neutrophil extracellular traps; NO, nitric oxide; Siglec, sialic acid-binding immunoglobulin-like lectin.

**Supplementary File 2.** Number of patients included in the study per assay

**
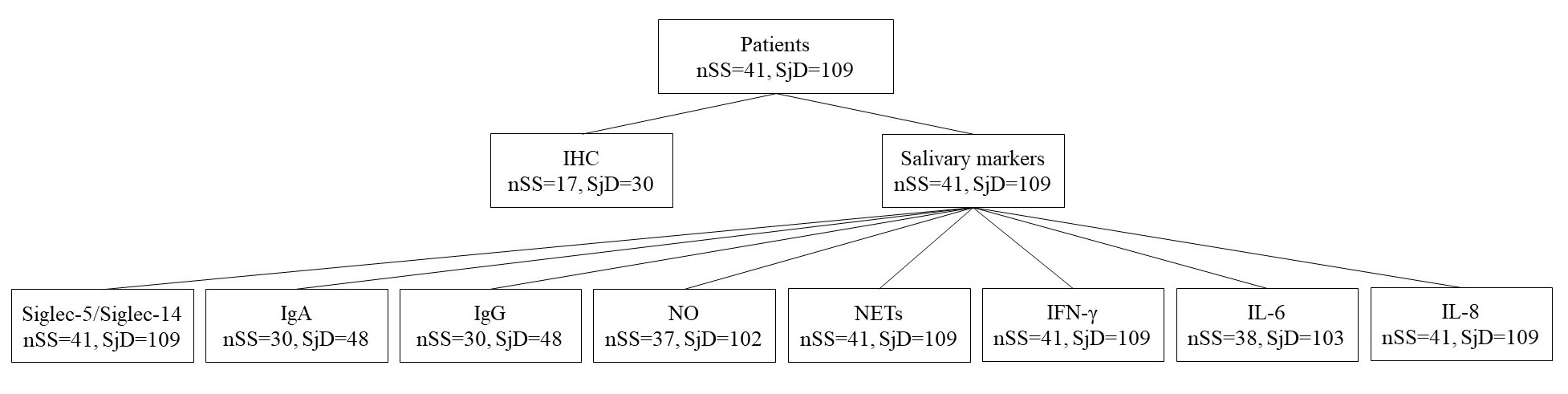
**

**Note:** IgA, immunoglobulin A; IgG, immunoglobulin G; IHC, immunohistochemistry; IL, interleukin; IFN, interferon; NETs, neutrophil extracellular traps; NO, nitric oxide; nSS, non-Sjögren *sicca*; Siglec, sialic acid-binding immunoglobulin-like lectin; SjD, Sjögren disease.

**Supplementary File 3.** Comparative analysis of comorbidity prevalence in patients with SjD and nSS

| Variables, n (%) | SjD (*n*=106) | nSS (*n*=41) | *p* value |
| --- | --- | --- | --- |
| RA |  |  | 0.446 ^ꝉ^ |
| Yes | 13 (12.3) | 7 (17.1) |  |
| No | 93 (87.7) | 34 (82.9) |  |
| SLE |  |  | 0.351^ǂ^ |
| Yes | 12 (11.3) | 2 (4.9) |  |
| No | 94 (88.7) | 39 (95.1) |  |
| Other rheumatological diseases |  |  | 0.390 ^ꝉ^ |
| Yes | 39 (36.8) | 12 (29.3) |  |
| No | 67 (63.2) | 29 (70.7) |  |
| Heart disease |  |  | 0.263 ^ꝉ^ |
| Yes | 60 (56.6) | 19 (46.3) |  |
| No | 46 (43.4) | 22 (53.7) |  |
| Metabolic diseases |  |  | 0.948 ^ꝉ^ |
| Yes | 29 (27.4) | 11 (26.8) |  |
| No | 77 (72.6) | 30 (73.2) |  |
| Respiratory diseases |  |  | 0.220^ǂ^ |
| Yes | 20 (18.9) | 4 (9.8) |  |
| No | 86 (81.1) | 37 (90.2) |  |
| Nervous system diseases |  |  | 0.337 ^ꝉ^ |
| Yes | 35 (33) | 17 (41.5) |  |
| No | 71 (67) | 24 (58.5) |  |
| Renal diseases |  |  | 0.656 ^ꝉ^ |
| Yes | 15 (14.2) | 7 (17.1) |  |
| No | 91 (85.8) | 34 (82.9) |  |
| Gastrointestinal diseases |  |  | 0.069 ^ꝉ^ |
| Yes | 19 (17.9) | 13 (31.7) |  |
| No | 87 (82.1) | 28 (68.3) |  |
| Thyroid diseases |  |  | **0.034^ǂ^** |
| Yes | 24 (22.6) | 3 (7.3) |  |
| No | 82 (77.4) | 38 (92.7) |  |
| Bone disease |  |  | 0.193 ^ꝉ^ |
| Yes | 29 (27.4) | 7 (17.1) |  |
| No | 77 (72.6) | 34 (82.9) |  |
| Malignant neoplasm |  |  | 1.000^ǂ^ |
| Yes | 3 (2.8) | 1 (2.4) |  |
| No | 103 (97.3) | 40 (97.6) |  |
| Lymphoma | 0 | 0 |  |
| Monoclonal gammopathy |  |  | 0.481^ǂ^ |
| Yes | 1 (0.9) | 1 (2.4) |  |
| No | 105 (99.1) | 40 (97.6) |  |

**Note:** nSS, non-Sjögren *sicca*; RA, rheumatoid arthritis; SjD, Sjögren disease; SLE, systemic lupus erythematosus.

^ǂ^Fisher exact test.

^ꝉ^Pearson Chi-Square test.

**Supplementary File 4.** Comparative analysis of medication use in patients with SjD and nSS

| Variables, n (%) | SjD (*n*=106) | nSS (*n*=41) | *p* value |
| --- | --- | --- | --- |
| Corticosteroid |  |  | 0.576^ꝉ^ |
| Yes | 28 (26.4) | 9 (22) |  |
| No | 78 (73.6) | 32 (78) |  |
| Hydroxychloroquine |  |  | 0.189^ǂ^ |
| Yes | 18 (17) | 3 (7.3) |  |
| No | 88 (83) | 38 (92.7) |  |
| Methotrexate |  |  | 0.656^ꝉ^ |
| Yes | 27 (25.5) | 9 (22) |  |
| No | 79 (74.5) | 32 (78) |  |
| Folic acid |  |  | 0.153^ꝉ^ |
| Yes | 24 (22.6) | 5 (12.2) |  |
| No | 82 (77.4) | 36 (87.8) |  |
| Mycophenolate mofetil |  |  | 0.322^ǂ^ |
| Yes | 5 (4.7) | 0 |  |
| No | 101 (95.3) | 41 (100) |  |
| Other immunosuppressants |  |  | 0.780^ǂ^ |
| Yes | 13 (12.3) | 4 (9.8) |  |
| No | 93 (87.7) | 37 (90.2) |  |
| Immunomodulators |  |  | 0.726^ǂ^ |
| Yes | 8 (7.5) | 2 (4.9) |  |
| No | 98 (92.5) | 39 (95.1) |  |
| Antihypertensives |  |  | 0.781^ꝉ^ |
| Yes | 57 (53.8) | 21 (51.2) |  |
| No | 49 (46.2) | 20 (48.8) |  |
| Thyroxine |  |  | **0.033^ǂ^** |
| Yes | 25 (23.6) | 3 (7.3) |  |
| No | 81 (76.4) | 38 (92.7) |  |
| Antidepressants |  |  | 0.587^ꝉ^ |
| Yes | 49 (46.2) | 21 (51.2) |  |
| No | 57 (53.8) | 20 (48.8) |  |
| Anticonvulsants |  |  | 0.870^ꝉ^ |
| Yes | 14 (13.2) | 92 (86.8) |  |
| No | 5 (12.2) | 36 (87.8) |  |
| Antipsychotics |  |  | 1.000^ǂ^ |
| Yes | 8 (7.5) | 3 (7.3) |  |
| No | 98 (92.5) | 38 (92.7) |  |
| Antidiabetics |  |  | 0.718^ꝉ^ |
| Yes | 18 (17) | 8 (19.5) |  |
| No | 88 (83) | 33 (80.5) |  |
| Statins |  |  | 0.640^ꝉ^ |
| Yes | 27 (25.5) | 12 (29.3) |  |
| No | 79 (74.5) | 29 (70.7) |  |
| Opioids |  |  | 0.671^ǂ^ |
| Yes | 4 (3.8) | 2 (4.9) |  |
| No | 102 (96.2) | 39 (95.1) |  |
| Anticoagulants |  |  | 1.000^ǂ^ |
| Yes | 5 (4.7) | 1 (2.4) |  |
| No | 101 (95.3) | 40 (97.6) |  |
| Bisphosphonates |  |  | 0.558^ǂ^ |
| Yes | 13 (12.3) | 3 (7.3) |  |
| No | 93 (87.7) | 38 (92.7) |  |
| Vitamin D |  |  | **0.004^ǂ^** |
| Yes | 31 (29.2) | 3 (7.3) |  |
| No | 75 (70.8) | 38 (92.7) |  |
| Calcium |  |  | 0.106^ꝉ^ |
| Yes | 32 (30.2) | 7 (17.1) |  |
| No | 74 (69.8) | 34 (82.9) |  |
| Eye drops |  |  | **0.026^ꝉ^** |
| Yes | 47 (44.3) | 10 (24.4) |  |
| No | 59 (55.7) | 31 (75.6) |  |

**Note:** nSS, non-Sjögren *sicca;* SjD, Sjögren disease.

^ǂ^Fisher exact test;

^ꝉ^Pearson Chi-Square test.

**Supplementary File 5.** Clinicodemographic data of SjD versus SjD overlapping with other rheumatic diseases

| Variables | SjD (*n=54*) | Overlapping SjD (*n=57*) | *p* value |
| --- | --- | --- | --- |
| Age, median (range) | 57 (10-76) | 58 (19-76) | 0.552^§^ |
| Sex, n (%) |  |  | 1.000^ǂ^ |
| Female | 49 (94.2) | 54 (94.7) |  |
| Male | 3 (5.8) | 3 (5.3) |  |
| Xerostomia, n (%) |  |  | 0.640^ꝉ^ |
| Yes | 38 (73.2) | 49 (86) |  |
| No | 8 (15.3) | 8 (14) |  |
| NR | 6 (11.5) | 0 |  |
| Xerophthalmia, n (%) |  |  | 0.151^ꝉ^ |
| Yes | 32 (61.5) | 48 (84.2) |  |
| No | 9 (17.3) | 6 (10.5) |  |
| NR | 11 (21.1) | 3 (5.2) |  |
| AntiRo/SSA, n (%) |  |  | **0.021 ^ꝉ^** |
| Positive | 31 (59.6) | 45 (78.9) |  |
| Negative | 18 (34.6) | 9 (15.8) |  |
| NR | 3 (5.7) | 3 (5.3) |  |
| Hyposalivation, n (%) |  |  | 0.594^ꝉ^ |
| Yes | 38 (73.1) | 39 (68.4) |  |
| No | 14 (26.9) | 18 (31.6) |  |
| Focus Score, median (range) | 2.3 (0-7.7) | 1.7 (0-7) | 0.095^§^ |
| Unstimulated whole salivary flow rate (ml/min), median (range) | 0.1 (0-0.9) | 0.1 (0-0.8) | 0.457^§^ |
| Parotitis, n (%) |  |  | 0.732^ꝉ^ |
| Yes | 22 (42.3) | 23 (40.3) |  |
| No | 25 (48.1) | 30 (52.6) |  |
| NR | 5 (9.6) | 4 (7.1) |  |

**Note:** NR, not reported; SjD, Sjögren disease.

^§^Mann-Whitney test.

^ǂ^Fisher exact test.

^ꝉ^Pearson chi-square test.

**Supplementary File 6.** Expression of inflammatory cells in SjD and nSS


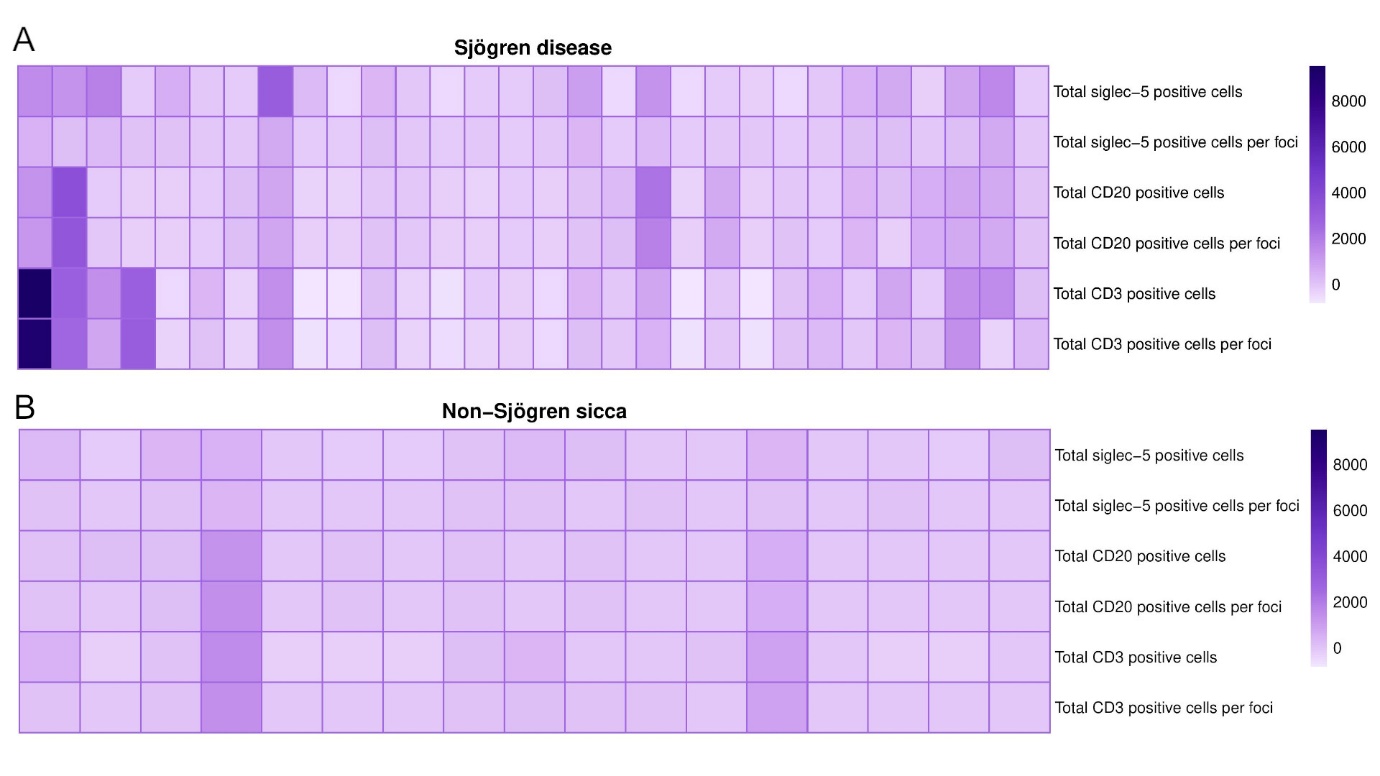


**Note:** Diagram illustrating differences in immunoquantification of siglec-5, CD3, and CD20 cells (total count and count per foci) between SjD and nSS groups. Each column represents an individual sample.

**Supplementary File 7.** Spearman correlation matrix of clinical parameters, salivary siglec-5/siglec-14 and inflammatory markers


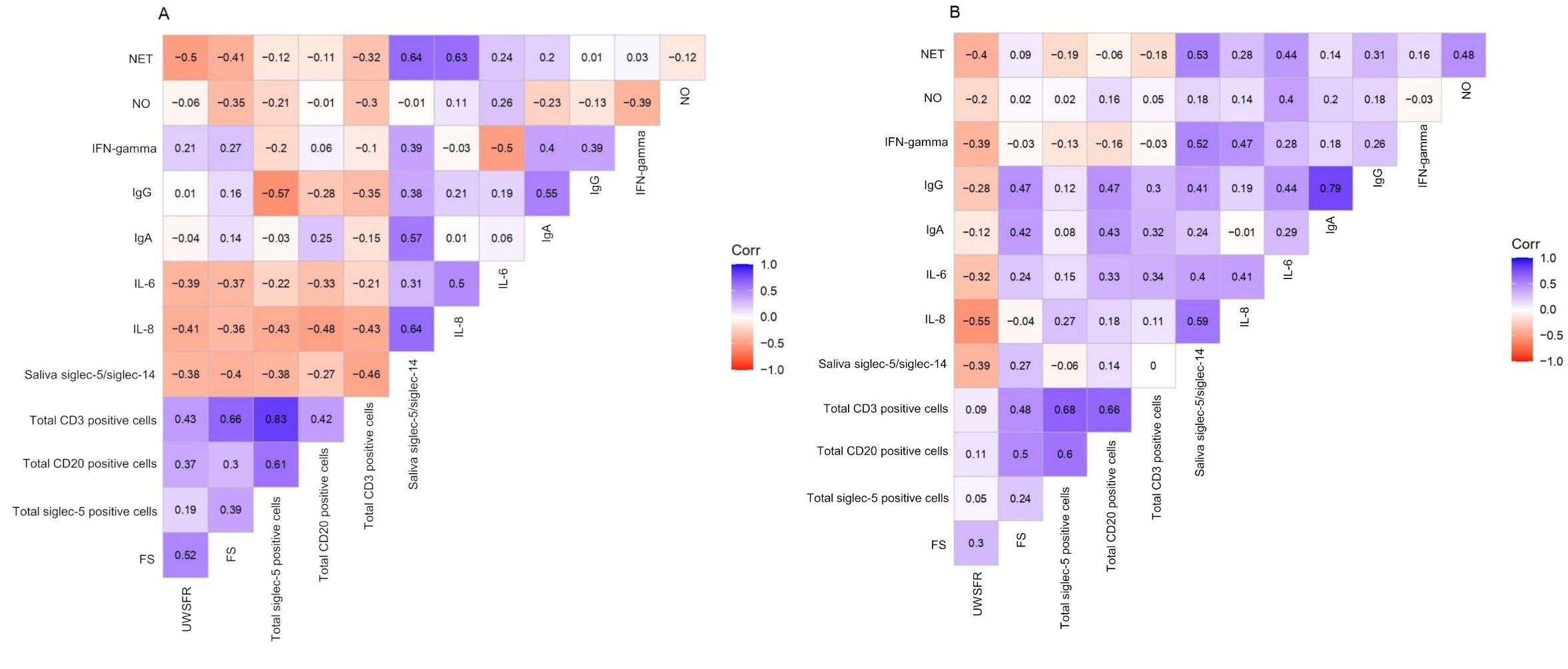


**Note:** Spearman correlation matrix showing pairwise associations. Values shown represent Spearman’s correlation coefficients (R). Positive correlations are indicated by positive R values, and negative correlations by negative R values. (A) nSS (B) SjD. SjD: Sjögren disease; nSS: non-Sjögren *sicca*; CODS: Clinical Oral Dryness Score; UWSFR: unstimulated salivary flow rate; FS: focus score; Siglec: Sialic acid-binding immunoglobulin-like lectins; IL: Interleukin.

| Variables^‡^ |  | Xerostomia | | |
| --- | --- | --- | --- | --- |
| Tissue |  | **Positive (*n*=27)** | **Negative (*n*=9)** | ***p* value** |
| Total siglec-5 positive cells | | 343 (0-3,511) | 343.5 (3-2,100) | 0.117^§^ |
| Total siglec-5 positive cells per foci | | 66 (0-776) | 0 (0-815) | **0.009^§^** |
| Total CD20 positive cells | | 150 (0-3,984) | 42 (0-1,064) | **0.029^§^** |
| Total CD20 positive cells per foci | | 53 (0-3,669) | 8 (0-952) | 0.348^§^ |
| Total CD3 positive cells | | 645 (0-10,334) | 103 (65-2,286) | 0.244^§^ |
| Total CD3 positive cells per foci | | 246 (0-9,739) | 35 (0-294) | 0.068^§^ |
| Saliva | | **Positive** | **Negative** |  |
| Siglec-5/Siglec-14, pg/mL (*n*=122 positive; *n*=21 negative) | | 1,626.9 (0-6,654.7) | 784.4 (17.7-1,192.3) | 0.736^§^ |
| IL-8, pg/mL (*n*=122 positive; *n*=21 negative) | | 995.5 (56.3-2,769.9) | 385.1 (146.6-752.1) | 0.714^§^ |
| IL-6, pg/mL (*n*=116 positive; *n*=19 negative) | | 9.4 (0.3-66.6) | 2.3 (0.2-16.5) | **0.037^§^** |
| IgA, ng/mL (*n*=65 positive; *n*=12 negative) | | 286.0 (85.5-1,492.1) | 327.2 (306.1-329.2) | 0.416^§^ |
| IgG, ng/mL (*n*=65 positive; *n*=12 negative) | | 17.8 (7.3-64.8) | 25.3 (19.9-28.5) | 0.573^§^ |
| IFN-γ, pg/mL (*n*=122 positive; *n*=21 negative) | | 19.7 (0-110.5) | 22.6 (9.9-33.9) | 0.414^§^ |
| NO, µM (*n*=117 positive; *n*=16 negative) | | 44.2 (8.7-325.1) | 24.7 (11.4-34) | **0.016^§^** |
| NETs, ng/mL (*n*=122 positive; *n*=21 negative) | | 36.3 (0-251.3) | 0 (0-17.9) | **0.005^§^** |

**Supplementary File 8.** Association of xerostomia with immune marker positivity (siglec-5, CD20 and CD3) and cytokine expression in salivary gland tissue and saliva

**Note:** FS, focus score; IgA, immunoglobulin A; IgG, immunoglobulin G; IL, interleukin; IFN, interferon; NETs, neutrophil extracellular traps; NO, nitric oxide; nSS, non-Sjögren *sicca*; Siglec, sialic acid-binding immunoglobulin-like lectin; SjD, Sjögren disease; UWSFR, unstimulated salivary flow rate.

^‡^Median and range.

^§^Mann-Whitney test.

**Supplementary File 9.** Evaluation of AUC, sensitivity, specificity, PPV, NPV, accuracy, Youden index, and cut-off point for 2016 EULAR classification in SjD

|  | AUC (%)  95% CI | SE for AUC | Sensitivity (%)  95% CI | Specificity (%)  95% CI | PPV (%)  95% CI | NPV (%)  95% CI | Accuracy (%) 95% CI | Youden index | Cut-off |
| --- | --- | --- | --- | --- | --- | --- | --- | --- | --- |
| Salivary siglec-5/siglec-14 (nSS=41, SjD=109) | 67 (58.9 – 74.4) | 0.0503 | 68.8 (59.2 – 77.3) | 58.5 (42.1 – 73.7) | 81.5 (75 – 86.6) | 41.3 (32.5 – 50.7) | 66 (57.8 – 73.5) | 0.2734 | >1984.54 |
| Tissue siglec-5 (nSS=17, SjD=30) | 73.1 (58.2 – 85) | 0.0734 | 46.6 (28.3 – 65.7) | 100 (80.5 – 100) | 100 (NaN – 100) | 51.5 (43.1 – 59.7) | 65.9 (50.6 – 79.1) | 0.4667 | >616 |
| Focus Score (nSS=40, SjD=94) | 89 (82.5 – 93.8) | 0.0333 | 86.1 (77.5 – 92.4) | 87.5 (73.2 – 95.8) | 94.1 (87.6 – 97.3) | 72.9 (61.5 – 81.8) | 86.5 (79.6 – 91.8) | 0.7367 | >0.94 |
| Anti-Ro/SSA (nSS=41, SjD=103) | 80.8 (73.4 – 86.9) | 0.0338 | 73.7 (64.2 – 82) | 87.8 (73.8 – 95.9) | 98.8 (86.9 – 97.2) | 57.1 (48.6 – 65.2) | 77.7 (70.1 – 84.2) | 0.6159 | - |
| Schirmer test (nSS=16, SjD=56) | 66.1 (54 – 76.8) | 0.0543 | 44.6 (31.3 – 58.5) | 87.5 (61.7 – 98.4) | 92.5 (76.8 – 97.9) | 31.1 (25.1 – 37.8) | 54.1 (42 – 65.9) | 0.3214 | - |
| Ocular Staining Score (nSS=16, SjD=57) | 72.7 (61 – 82.5) | 0.0540 | 57.8 (44.1 – 70.9) | 87.5 (61.7 – 98.4) | 94.3 (81.5 – 98.4) | 36.8 (29 – 45.4) | 64.3 (52.3 – 75.2) | 0.4539 | - |

**Note:** AUC: area under the curve; CI: confidence interval; NaN: not a number; NPV: negative predictive value; nSS: non-Sjögren sicca; PPV: positive predictive value; SE: standard error; Siglec, sialic acid-binding immunoglobulin-like lectin; SjD: Sjögren disease.

**Supplementary File 10.** Pairwise AUC comparisons (DeLong’s test)

|  | 95% Confidence Interval | | | | |
| --- | --- | --- | --- | --- | --- |
|  | **AUC difference** | **Lower** | **Upper** | **z** | **p** |
| Salivary siglec-5 vs. tissue siglec-5 | -0.06142 | -0.336 | 0.15577 | -0.7187 | 0.472 |
| Salivary siglec-5 vs. Anti-Ro/SSA | -0.13801 | -0.255 | -0.02061 | -2.3040 | 0.021 |
| Salivary siglec-5 vs. Schirmer test | 0.00923 | -0.139 | 0.23755 | 0.5108 | 0.610 |
| Salivary siglec-5 vs. OSS | -0.05703 | -0.201 | 0.17686 | -0.1251 | 0.900 |
| Salivary siglec-5 vs. FS | -0.22048 | -0.358 | -0.10716 | -3.6329 | <0.001 |
| Tissue siglec-5 vs. Anti-Ro/SSA | -0.07658 | -0.190 | 0.15271 | -0.2131 | 0.831 |
| Tissue siglec-5 vs. Schirmer test | 0.07066 | -0.625 | 0.79186 | 0.2305 | 0.818 |
| Tissue siglec-5 vs. OSS | 0.00440 | -0.462 | 0.29493 | -0.4318 | 0.666 |
| Tissue siglec-5 vs. FS | -0.15905 | -0.305 | 6.85e-4 | -1.9512 | 0.051 |
| Anti-Ro/SSA vs. Schirmer test | 0.14724 | -0.141 | 0.21759 | 0.4190 | 0.675 |
| Anti-Ro/SSA vs. OSS | 0.08098 | -0.181 | 0.17503 | -0.0313 | 0.975 |
| Anti-Ro/SSA vs. FS | -0.08247 | -0.206 | 0.00169 | -1.9281 | 0.054 |
| Schirmer test vs. OSS | -0.06626 | -0.185 | 0.06776 | -0.9108 | 0.362 |
| Schirmer test vs. FS | -0.22971 | -0.355 | 0.05667 | -1.4207 | 0.155 |
| OSS vs. FS | -0.16345 | -0.261 | 0.15080 | -0.5261 | 0.599 |

**Note:** AUC: area under the curve; FS: focus score; OSS: ocular staining score; Siglec, sialic acid-binding immunoglobulin-like lectin.

**Supplementary File 11.** Combined ROC curves


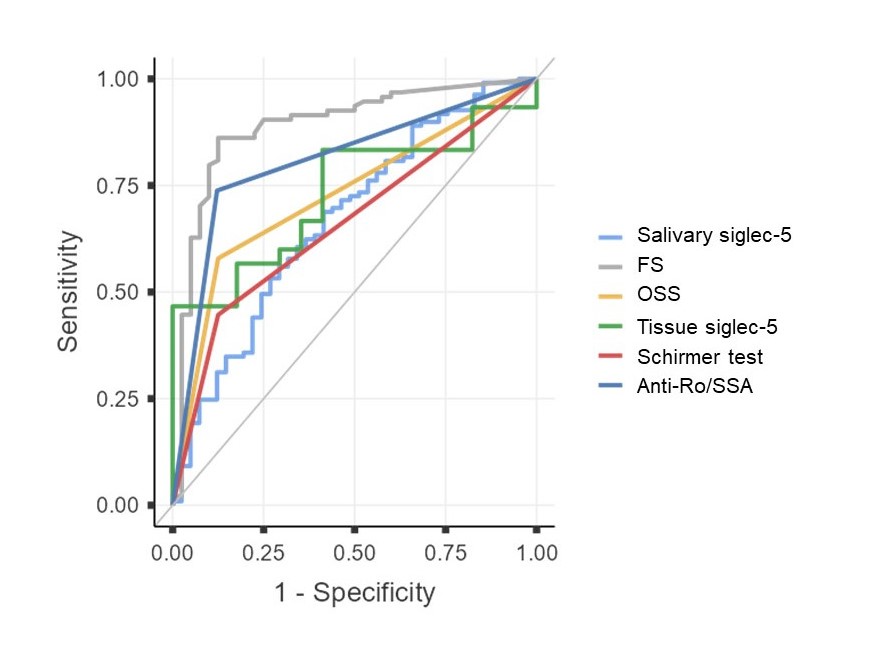


**Note:** FS: focus score; OSS: ocular staining score; ROC: Receiver Operating Characteristic; Siglec, sialic acid-binding immunoglobulin-like lectin.
